# Univariate and multivariate spatial models of health facility utilisation for childhood fevers in an area on the coast of Kenya

**DOI:** 10.1186/s12942-017-0107-7

**Published:** 2017-09-18

**Authors:** Paul O. Ouma, Nathan O. Agutu, Robert W. Snow, Abdisalan M. Noor

**Affiliations:** 1 Department of Geomatic Engineering and Geospatial Information Systems, Jomo Kenyatta University of Agriculture and Technology, Nairobi, Kenya; 2 Kenya Medical Research Institute/Wellcome Trust Research Programme, Nairobi, Kenya; 3 Centre for Tropical Medicine and Global Health, Nuffield Department of Clinical Medicine, University of Oxford, Oxford, UK

**Keywords:** Utilisation, Fever, Univariate, Multivariate

## Abstract

**Background:**

Precise quantification of health service utilisation is important for the estimation of disease burden and allocation of health resources. Current approaches to mapping health facility utilisation rely on spatial accessibility alone as the predictor. However, other spatially varying social, demographic and economic factors may affect the use of health services. The exclusion of these factors can lead to the inaccurate estimation of health facility utilisation. Here, we compare the accuracy of a univariate spatial model, developed only from estimated travel time, to a multivariate model that also includes relevant social, demographic and economic factors.

**Methods:**

A theoretical surface of travel time to the nearest public health facility was developed. These were assigned to each child reported to have had fever in the Kenya demographic and health survey of 2014 (KDHS 2014). The relationship of child treatment seeking for fever with travel time, household and individual factors from the KDHS2014 were determined using multilevel mixed modelling. Bayesian information criterion (BIC) and likelihood ratio test (LRT) tests were carried out to measure how selected factors improve parsimony and goodness of fit of the time model. Using the mixed model, a univariate spatial model of health facility utilisation was fitted using travel time as the predictor. The mixed model was also used to compute a multivariate spatial model of utilisation, using travel time and modelled surfaces of selected household and individual factors as predictors. The univariate and multivariate spatial models were then compared using the receiver operating area under the curve (AUC) and a percent correct prediction (PCP) test.

**Results:**

The best fitting multivariate model had travel time, household wealth index and number of children in household as the predictors. These factors reduced BIC of the time model from 4008 to 2959, a change which was confirmed by the LRT test. Although there was a high correlation of the two modelled probability surfaces (Adj *R* ^2^ = 88%), the multivariate model had better AUC compared to the univariate model; 0.83 versus 0.73 and PCP 0.61 versus 0.45 values.

**Conclusion:**

Our study shows that a model that uses travel time, as well as household and individual-level socio-demographic factors, results in a more accurate estimation of use of health facilities for the treatment of childhood fever, compared to one that relies on only travel time.

**Electronic supplementary material:**

The online version of this article (doi:10.1186/s12942-017-0107-7) contains supplementary material, which is available to authorized users.

## Background

Health facility utilisation is an important metric for understanding the uptake of health services [1, 2], quantification of health commodities and estimation of disease burden [3–5]. Developing spatial models of utilisation has been the subject of much research, ranging from the use of Euclidean distances, to more sophisticated travel time models as proxies of utilisation [6]. Improvements to these models include the use of individual level attendance patterns, in conjunction with the accessibility surfaces to define utilisation at high spatial resolutions [1, 2, 7, 8]. Traditionally, spatial models account for the influence of physical distances alone, ignoring other demographic characteristics that affect decisions to use a health facility [9]. As health information systems improve in low-income countries [10], and the use of routine data for the estimation of burden of diseases and quantification of health resources increase, the need for accurate models of utilisation also increase [3, 4]. Therefore, developing a greater understanding of the role of non-spatial factors that influence the treatment seeking behaviours of febrile individuals [11] become crucial in spatial modeling of utilisation.

Fever is one of the most common reasons for seeking care at health facilities in Africa, especially for children under 5 years [12]. Thus, treatment seeking for fever has been used to map the variation in utilisation, using univariate models [1]. However, other factors such as maternal education [13], wealth [14, 15], and severity of illness [16, 17], also affect utilisation of health services for fever. Conceptually, therefore, the exclusion of these additional covariates into the classical spatial only health facility utilisation models is likely to provide an incomplete picture. Bypassing these variables can be attributed to lack of data, but with availability of interpolation techniques such as kriging, mapping these variables at fine spatial resolutions becomes possible. In addition, as geocoded data on childhood treatment seeking in sub-Saharan Africa become increasingly available, it is now possible to explore the added benefits of multivariate utilisation models over the standard univariate spatial-only models.

In this study, we compare two utilisation models: one developed using a probability of attendance computed from the results of a univariate logistic regression model with travel time as the dependent variable, and the other from a multivariate model with travel time and selected socio-demographic covariates as dependent variables. In both models, analysis is restricted to the use of public health facilities, which can be easily mapped [18], and whose spatial dimension of access commonly matters in resource constrained settings. We then compare the parsimony, goodness of fit, discriminatory characteristics and goodness of fit of the two models using Bayesian information criterion (BIC), likelihood ratio test (LRT), receiver operating area under the curve (AUC) and a percent correct prediction test (PCP) respectively.

## Methods

### Study area

The study area encompasses Kilifi, Kwale and Mombasa counties on the coast of Kenya covering approximately 21,000 km^2^. This area is generally flat, with elevation rising from sea level along the coastline to a maximum of approximately 850 m above mean sea level. Over 60% of the population lives in rural areas, while majority of the urban population reside in the three major urban centres of Mombasa, Kilifi and Malindi towns, which are all on the shores of Indian Ocean. The three counties are considered to be malaria endemic [19], with variable socio-demographic characteristics, providing an ideal setting for conducting this study. Treatment seeking rates for fever among children under the age of 5 years ranged between 63 and 75% in the three counties [20].

### Data and sources

Different sources were used to develop a health facility database including: the ministry of health master facility list; Development Partners for Health in Kenya [21]; the district health information system [22]; and published sources [18]. These databases were merged using names and master facility codes. While majority had latitudes and longitudes, online sources such as Google earth [23] and OpenStreetMaps [24] were used for geolocating facilities without coordinates. Finally, the coordinates were checked by mapping onto Google earth, and those falling in unlikely places such as water bodies, forests and roads were shifted to structures where they were likely to be. The final list had names of facilities, codes, levels of care, spatial information and facility type as the attributes.

The Kenya Demographic Health Survey (KDHS 2014) was the largest national household survey in Kenya, conducted as part of a series of surveys for monitoring population health status, with the aim of having most of them statistically powered to provide estimates at county level [20]. The survey was based on a stratified two-stage sampling technique, where in the first stage, clusters in either rural or urban areas were selected. In the second stage, approximately 25 households were randomly selected from each cluster, and visited for the interviews. The GPS coordinates of the clusters were also recorded. Treatment seeking for fever among children under 5 years was one of the indicators collected in the DHS.

Attendance at public facilities for the two week history of febrile illnesses for children under 5 years, was used as the outcome variable. Public health facilities in Kenya are those managed by the government, faith-based institutions and other non-profit organizations [25]. A literature search on other socio-demographic factors likely to affect health facility utilisation was conducted as shown in the Additional file 1. Those available in the DHS were determined and extracted in the DHS.

Land cover data was obtained from the GlobeLand30 project, developed from Landsat imagery of 2010 at 30 m spatial resolution [26]. Regional surfaces of Kenya Digital Elevation Models were downloaded from the USGS land processes distributed active archive centre (LP DAAC) website, at 30 m spatial resolution [27]. Freely available road network datasets from OpenStreetMaps [24] were downloaded, and updated using georeferenced Kenya roads board maps at 1:25,000 scale [28]. Population surfaces were obtained from the WorldPop database [29].

### Analysis

#### Spatial access to health facilities

Travel speeds on various travel surfaces, in kilometre per hour, were assembled from various sources [1, 30]. These were assigned to the various types of roads and land cover surfaces. Impedance values, defined as the degree to which a surface is a barrier to travel, were assigned to major water features, changing elevation, and dense forests. A *cost friction surface*, which is a measure of the speeds and impedance across various features, was generated. A combination of the health facilities and the cost friction surface was used to model travel time to the nearest public health facility, using the *path distance* tool in ArcGIS 10.1 (ESRI, Inc., Redland, CA, USA). The travel times were extracted to each cluster location and assigned to every child who had fever in the last 2 weeks. This was used to estimate the time required for each child to get to the nearest public health facility.

#### Models of health facility utilisation

Previously, a three parameter logistic regression (3PL), with travel time as the predictor, has been used to model health facility utilisation [1]. The 3PL model uses item response theory, which includes a probability parameter of a positive response given binary input variables as a logistic distribution [31]. Here, we propose the use of a generalised linear mixed effects model (GLMM) that combines the properties of both generalised linear models (GLMs including logistic regressions) and linear mixed models (which include random effects). This model was chosen because data from household surveys often have a clustered structure. Thus, the model treats clustered data adequately, assuming inter- and intra-cluster variation with the model specified in hierarchical fashion [31]. The model, also includes the random effects, which account for unobserved variables that may affect the outcome. Classical logistic regressions on the other hand, assume that observations are identically distributed and independent, an assumption which may be unrealistic.

The GLMM models the probability that a child *i* living in cluster *j,* will attend a public health facility, given by;

$$\log it\left\{ {P(Y = 1|(x_{1} ,x_{2} , \ldots ,x_{n} ),\sigma_{c} } \right\} = c_{0} + c_{1} * x_{1ij} + c_{2ij} * x_{2ij} + \cdots + c_{n} * x_{nij} + \sigma_{cj} \sigma_{c} \sim N(0,\tau^{2} )$$

where $x_{1} ,x_{2} ,x_{3} , \ldots ,x_{n}$ were the variables of interest. $c_{0}$ was the overall intercept of the model while $c_{1}$ to $c_{n}$ the specific variable coefficients. Y was the attendance versus non-attendance for fever pattern. $\sigma_{cj}$ was the random effect assigned at the cluster level. The random effects were assigned to account for community level information such as cultural practices that may not have been captured in the DHS but affect decision or ability to attend a health facility.

Before model selection, 10% of the data was randomly selected for use in the validation exercise. The initial model had travel time, household wealth index, maternal education, residence and number of children in household as the predictor variables. Bi-variable correlations were analysed using a threshold of 75% to remove problems of multicollinearity in the model. Residence highly correlated (90%) with wealth quintiles, but the residence variable dropped because of a lower BIC value in univariate models.

While holding travel time constant, the additional variables were added sequentially without replacement to the model. In each iteration, the variable giving the lowest Bayesian information criterion (BIC) was chosen. To highlight the influence of additional variables on goodness of fit, a likelihood ratio test was carried out between the GLMM model with time as predictor and the final multivariate model. The analysis was carried out in R statistical software version 3.0, using the *lme4* package [32]. As wealth can affect attendance across different travel times and households with different number of children, interaction effects were included, between wealth:children and wealth:travel time. To estimate catchment areas, a distance decay curve was plotted using travel time as the predictor. To highlight the influence of the selected covariates on distance decay curve, the model was fitted to households with one or no child (≤1) and those with more than one child (>1). In the second case, the model was fitted to different groups of wealth quintiles (1, 2) and (3, 4, 5).

#### Spatial interpolation of the variables

To predict poverty and household number of children, we used ordinary kriging. Kriging is a generic term for probabilistic models that use the weighted sum of observed values, to provide best linear unbiased predictions [33]. Proportions of the two classes; ≤1 child and wealth quintiles (1, 2) were then calculated and spatial autocorrelation explored by plotting a semivariogram for each of the two datasets. The interpolation process exploited this spatial dependency, to generate estimates of the variables at all locations in the study area Additional file 1. This was implemented in R v3.0 statistical, using the *geoR* package with predictions at 100 m spatial resolution.

The coefficients of the time model were applied to the travel time alone (univariate model) and then to travel time and modelled surfaces of poverty and number of children in households (multivariate model) to generate two spatial models of utilisation.

### Comparing the utilisation models

The probabilities of health facility utilisation from the two models were extracted to each child location in the validation dataset for comparison. First, the relationship of the two values was assessed using the adjusted *R* ^2^ statistic. Secondly, the receiver operating area under the curve (AUC) was fitted to the two model outputs. The area defined by this curve shows the probability that a randomly selected attendance outcome is correctly predicted than a randomly chosen non-attendance value [34]. To check for robustness of both models, the process was repeated two more times with different selected validation datasets. Thirdly, comparison was done using the ‘*percent correct prediction*’ (PCP) test. This method is commonly used in analysis of the accuracy of predicted landslide suitability maps generated from logistic regressions [35]. In this exercise, the validation dataset was used to test both models. A correct prediction of probability of attendance was assumed if the probability value was greater than 0.5 and the child attended or less than 0.5 and the child did not attend. The opposite result was recorded as an incorrect prediction and 0.5 indicative of model failure. This was done for the two predicted surfaces and the final percent correct predictions for the two models determined and compared. A sensitivity analysis was carried out where the accuracy assessment was undertaken with probability cut off values of 0.40, 0.45, 0.55 or 0.60 Additional file 1. To highlight the influence of using either of the models in estimating the fever burden, model outputs were multiplied with population surfaces at catchment areas to define the catchment population. These populations were then multiplied by fever prevalence surfaces to estimate febrile children likely to attend facilities. The results were extracted at catchment areas and multiplied by 26 to estimate all fever cases in 2014.

## Results

**Fig. 1:**
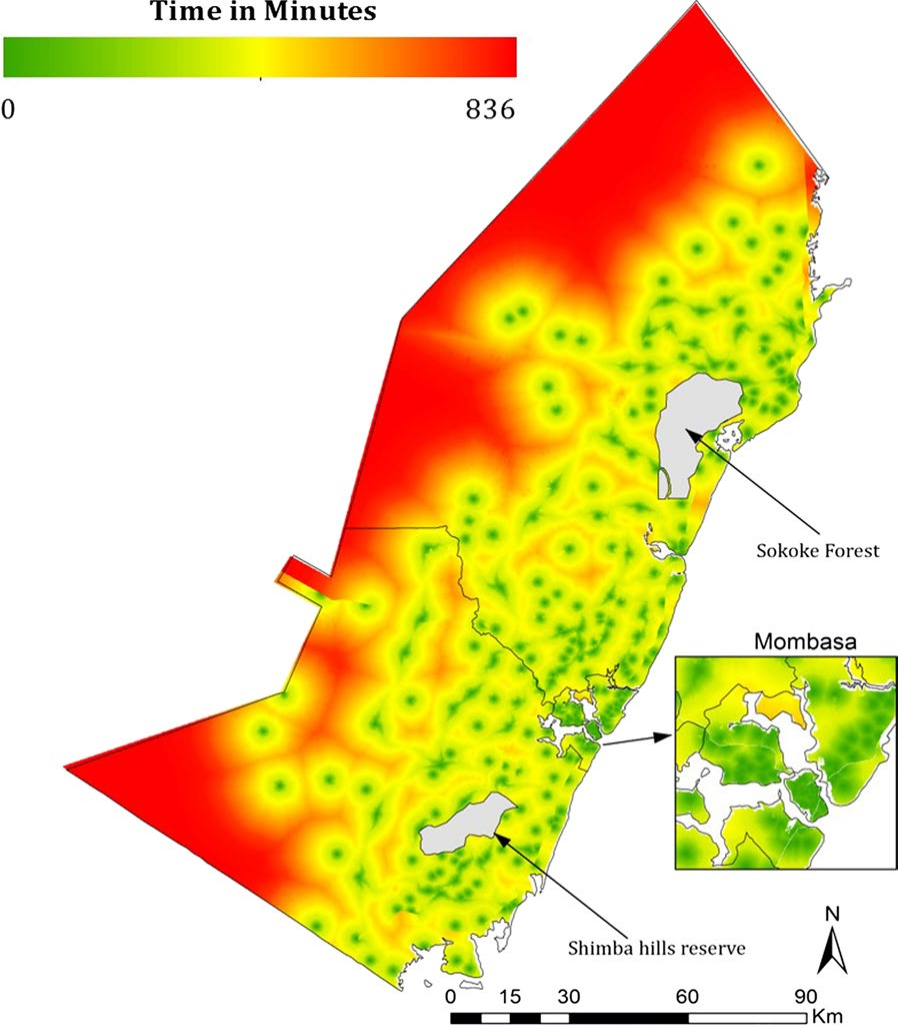
Travel time to the nearest health facility, ranging from zero (green) to 836 min (red). The protected areas are shown in grey

The study area had 311 public health facilities that were all geo-located. A sample of 345 children, representing a 26.0% [95% CI 22.5–30.0] prevalence of fever was used. Of these children, 79.8% [95% CI 74.5–84.2] sought treatment from an appropriate provider, while 57% [95% CI 49.8–63.9] sought treatment from public health facilities. Estimated travel time to public health facilities varied across the study area with the highest estimates being approximately 836 min as shown in Fig. 1.

**Fig. 2:**
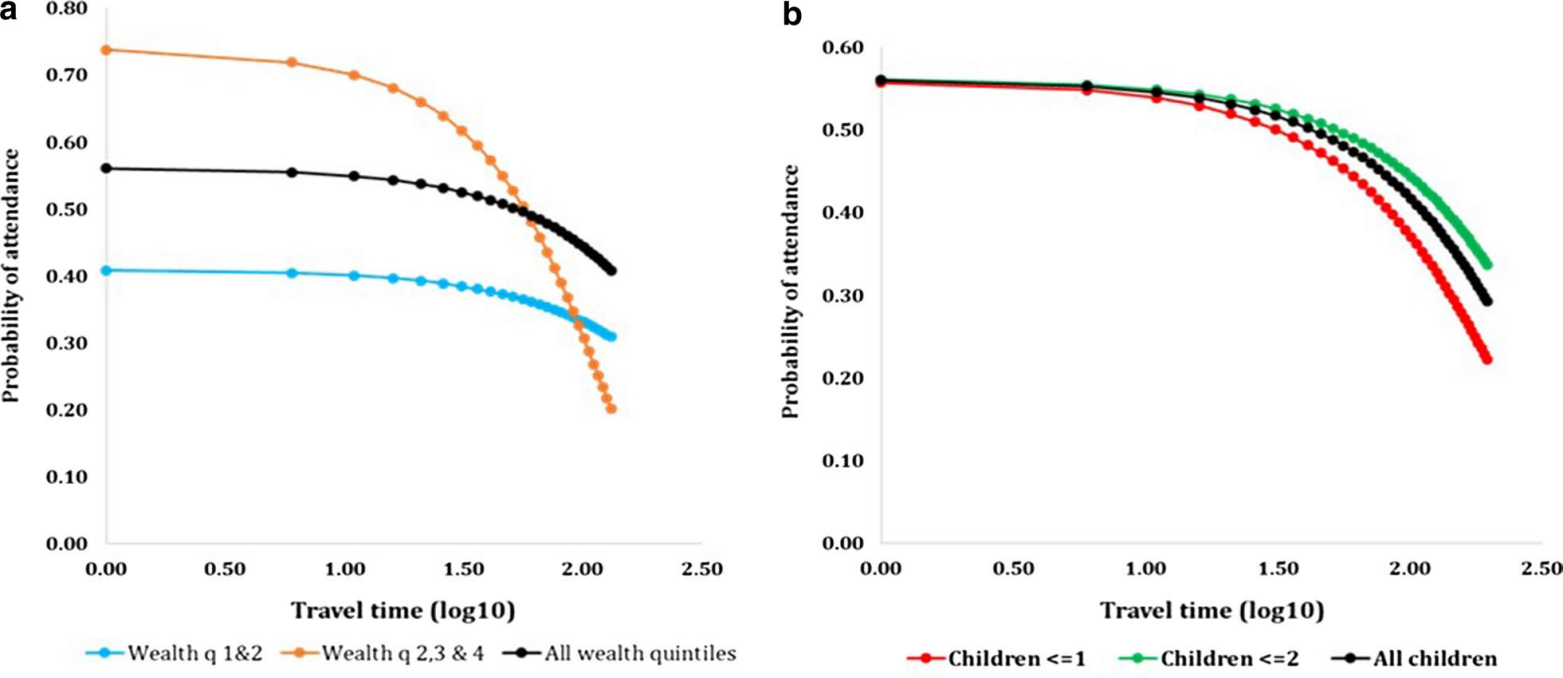
Modelled decay curves for different classes of **a** wealth quintiles where the blue curve represents lower wealth quintiles and the orange curve the higher wealth quintiles. **b** Shows number of children in a household, where red represents those in households with at most one child and the green curve those coming from households with at least two children. The black curves show the overall model fit without classification

The distance decay curve generated from the univariate model, showed rapid decay of facility attendance after about 51 min, as shown in Fig. 2a, b. The black curves show the overall curves fitted without classification. However, when the distance decay curves were plotted for different classes of household wealth and number of children, they significantly varied as shown in Fig. 2a, b. Children in the higher wealth quintiles (orange curve) had higher probabilities of attendance at close proximity to public health facilities. These probabilities, however, decayed more rapidly compared to those in the lower wealth quintiles. Distance decay also varied with number of children in a household, and although they were generally similar at close proximity to public facilities, variation was visibly significant as travel time increased.

The multivariate model had travel time, wealth quintile and number of children in a household as the significant predictors of health facility utilisation. Generally, households with lower socio economic status were found to have higher chances of attending public health facilities (OR 2.549). In addition, having a higher number of children in a household reduced the probability of public health facility attendance (OR 0.664). All the model coefficients including interaction terms were statistically significant, and are summarised in Table 1.

**Table 1:** Mixed model coefficients showing the relationship between attendance and the predictor variables, with a variance of random effects of 9.6

	Variables	Coefficient	Odds ratio	SE	*p* value
	Overall intercept	−0.166	0.846	0.001	<0.001
Fixed effects	Travel time	−0.017	0.983	0.001	<0.001
	Wealth (poorer and poorest)	1			
	Wealth (middle, richer and richest)	0.936	2.549	0.001	<0.001
	Number of children (≤1)	1			
	Number of children (>1)	−0.410	0.664	0.001	<0.001
Interaction terms	Travel time and wealth	−0.005	0.995	0.001	<0.001
	Wealth and number of children	1.326	3.766	0.001	<0.001

**Fig. 3:**
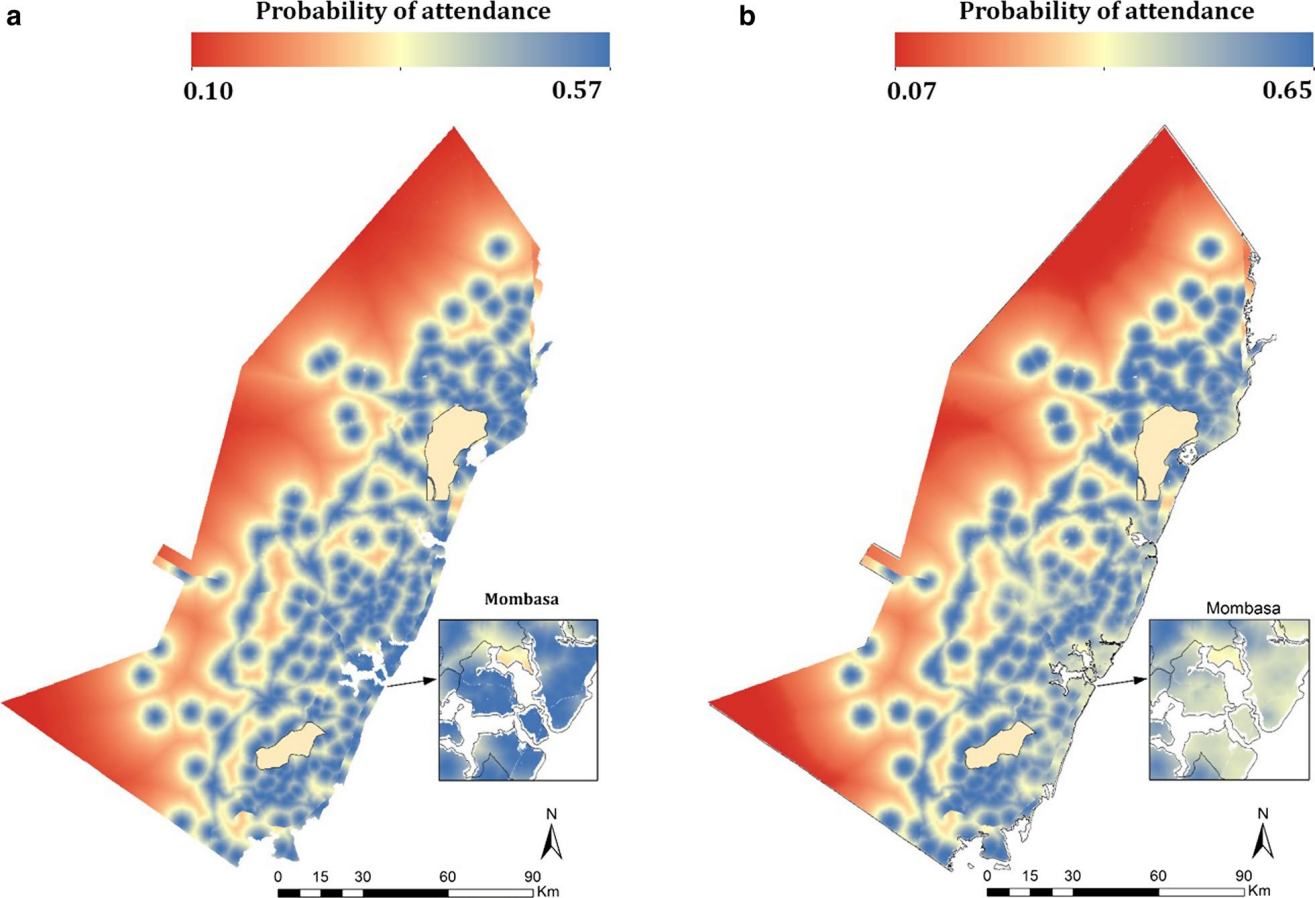
Public health facility utilisation maps. **a** From travel time alone and **b** from multivariate model which includes the wealth and number of children in household as predictors

Two scenarios of public health facility attendance were produced, one based on the univariate model and the other on the multivariate comparator. In the univariate model, the highest probabilities were recorded in areas close to health facilities where probability was approximately 52%. The cell with the lowest probability was in remote areas where travel times to health facilities were highest. In the multivariate model, the highest probabilities were recorded in rural areas close to health facilities where probability was approximately 65%. The areas with lowest probabilities were in remote areas where travel times tended to be high, as shown in Fig. 3. An intuitive outcome was that probability of attending public health facilities in highly accessible and urbanized areas such as Mombasa, was much lower in the multivariate model compared to those observed in the univariate model as shown Fig. 3.

The two models were highly correlated, with an adjusted *R* ^2^ value of 88%. The BIC values consistently reduced with addition of more covariates into the model, indicating that accuracy of the model was increasing. The best fitting model was determined as the one with time, wealth and number of children in a household. The model had a BIC of 4008 a value which reduced to 2959 with the introduction of interaction terms. The LRT test showed that goodness of fit of the multivariate model was better than that of the time model. In comparing the predictive capabilities of the two models, the univariate model had an AUC value of 0.73, while the multivariate model had an AUC value of 0.82. When the accuracy was checked again using two randomly selected validation data, the AUC values were 0.69 versus 0.73 and 0.68 versus 0.80 for the univariate and multivariate models respectively. The final predictions of health facility attendance were compared to the subset data initially created with the 10% hold-out set. With respect to PCP test, model 1 gave a value of 0.45 while model 2 gave a value of 0.61. According to univariate model, an estimated 1.76 million fever cases for children under 5 years were expected to be presented at public health facilities. On the other hand, the multivariate model, estimated 1.34 million fever cases, a 21% reduction.

## Discussion

Accurate estimation of the use of health services is critical to the precise quantification of several health metrics, including disease burden and commodity needs [3–5]. In this study, we compared the accuracy of the commonly used univariate spatial model of utilisation which has spatial access (travel time) as the sole predictor, to a multivariate model which also includes other social, demographic and economic factors that are important to treatment seeking [10, 14, 15]. To our knowledge, this was the first attempt towards creating a multivariate spatial model of utilisation at fine spatial resolution. The additional factors generally improved the parsimony and goodness of fit of the time model. This study highlights the importance of using additional covariates in modelling facility use. This approach is likely to provide a better understanding of variation in health facility utilisation, estimation of disease burden and quantifying resources.

Parsimony is an important characteristic of statistical models, and in our case, the additional covariates had a superior explanatory power, compared to the time alone model. The likelihood ratio test (LRT) also showed that the multivariate model had a superior goodness of fit. Thus, the null hypothesis that the time model was a better predictor of public facility utilisation was rejected. Testing the model performance using a separate dataset, the predictive capability of the univariate model was lower (AUCs = 0.73, 0.69 and 0.68), compared to that of the multivariate model (AUC = 0.83, 0.73 and 0.80). This indicates that the multivariate model was better at discriminating between attendance and non-attendance, compared to the univariate model [36]. The percent correct prediction test also displayed improved model prediction capabilities from the univariate to the multivariate model, even by using different cut off values for the assessment.

Two examples highlight the importance of the additional covariates in our study. First, in the univariate model where travel time was the sole predictor, probability of use was high in Mombasa where public health facilities are close. However, the multivariate model had much reduced probabilities when wealth index, maternal education and household size were added. This result is consistent with those of the Kenya health expenditure and utilisation survey, which analysed treatment seeking patterns at household levels, and showed that urban populations are less likely than rural populations to visit public health facilities [37]. Second, the estimation of the fever cases expected to attend public health facilities in 2014, at catchment areas also varied, with the multivariate model showing a 21% reduction in the burden of fever presented at health facilities.

In addition to spatial, social, demographic and economic factors, utilisation can be affected by seasonality as well as quality of services provided, both of which are unaccounted for in our model due to lack of data. Furthermore, the probability of health service utilisation in our analysis is inferred to mean use of the nearest health facility given the household survey data did not have information of the actual facility that was used. Finally, treatment seeking could be variable by the type of disease and the expected services at point of care. Depending on the severity of other symptoms accompanying an episode of fever, guardians of a child may seek care from different levels of the health facility at different times as well. Such decision pathways and resultant heterogeneity require a more detailed population level study and are outside the scope of our analysis.

## Conclusion

Our study demonstrates that commonly used univariate spatial models of health service utilisation perform poorly relative to multivariate models that include selected social, demographic and economic factors that determine treatment seeking. Additional covariates improved the overall model parsimony and goodness of it. The analysis also highlights potential implications in differences in urban and rural treatment seeking and estimation of burden of fever.

## Additional file

**Additional file 1.** Additional file on the determinats of health facility utilisation and detailed kriging methods used for interpolation of covariates.

## Abbreviations

AUC: area under the curve
LRT: likelihood ratio test
BIC: Bayesian information criterion
GIS: geographic information system

## References

[1] Alegana VA, Wright JA, Pentrina U, Noor AM, Snow RW, Atkinson PM (2012). Spatial modelling of healthcare utilisation for treatment of fever in Namibia. Int J Health Geogr..

[2] Noor AM, Amin AA, Gething PW, Atkinson PM, Hay SI, Snow RW (2006). Modelling distances travelled to government health services in Kenya. Trop Med Int Health.

[3] Alegana VA, Wright JA, Nahzat SM, Butt W, Sediqi AW, Habib N (2014). Modelling the incidence of *Plasmodium vivax* and *Plasmodium falciparum* malaria in Afghanistan 2006–2009. PLoS ONE.

[4] Alegana VA, Atkinson PM, Wright JA, Kamwi R, Uusiku P, Katokele S (2013). Estimation of malaria incidence in northern Namibia in 2009 using Bayesian conditional-autoregressive spatial-temporal models. Spat Spatiotemporal Epidemiol.

[5] Sturrock HJW, Cohen JM, Keil P, Tatem AJ, Le Menach A, Ntshalintshali NE (2014). Fine-scale malaria risk mapping from routine aggregated case data. Malar J.

[6] Neutens T (2015). Accessibility, equity and health care: review and research directions for transport geographers. J Transp Geogr.

[7] Tanser F, Gijsbertsen B, Herbst K (2006). Modelling and understanding primary health care accessibility and utilization in rural South Africa : an exploration using a geographical information system. Soc Sci Med.

[8] Ruktanonchai CW, Ruktanonchai NW, Nove A, Lopes S, Pezzulo C, Bosco C (2016). Equality in maternal and newborn health: modelling geographic disparities in utilisation of care in five east african countries. PLoS ONE.

[9] Ensor T, Cooper S (2004). Overcoming barriers to health service access: influencing the demand side. Health Policy Plan.

[10] Mutale W, Chintu N, Amoroso C, Awoonor-Williams K, Phillips J, Baynes C (2013). Improving health information systems for decision making across five sub-Saharan African countries: implementation strategies from the African Health Initiative. BMC Health Serv Res.

[11] Novignon J, Nonvignon J (2012). Socioeconomic status and the prevalence of fever in children under age five: evidence from four sub-Saharan African countries. BMC Res Notes.

[12] World Health Organisation (WHO). World Health Statistics. 2013. http://apps.who.int/gho/data/node.main.ChildMortDistRegion?lang=en. Accessed Sept 2016.

[13] Kanté AM, Gutierrez HR, Larsen AM, Jackson EF, Helleringer S, Exavery A (2015). Childhood illness prevalence and health seeking behavior patterns in rural Tanzania. BMC Public Health.

[14] Diaz T, George AS, Rao SR, Bangura PS, Baimba JB, McMahon SA (2013). Healthcare seeking for diarrhoea, malaria and pneumonia among children in four poor rural districts in Sierra Leone in the context of free health care: results of a cross-sectional survey. BMC Public Health.

[15] Ellis AA, Traore S, Doumbia S, Dalglish SL, Winch PJ (2012). Treatment actions and treatment failure: case studies in the response to severe childhood febrile illness in Mali. BMC Public Health.

[16] Ustrup M, Ngwira B, Stockman LJ, Deming M, Nyasulu P, Bowie C (2014). Potential barriers to healthcare in malawi for under-five children with cough and fever: a national household survey. J Health Popul Nutr.

[17] Webair HH, Bin-Gouth AS (2013). Factors affecting health seeking behavior for common childhood illnesses in Yemen. Patient Prefer Adher.

[18] Noor AM, Alegana VA, Gething PW, Snow RW (2009). A spatial national health facility database for public health sector planning in Kenya in 2008. Int J Health Geogr.

[19] Noor AM, Gething PW, Alegana VA, Patil AP, Hay SI, Muchiri E (2009). The risks of malaria infection in Kenya in 2009. BMC Infect Dis.

[20] Kenya National Bureau of Statistics (KNBS), ICF International. Kenya Demographic and Health Survey 2014. 2015. https://dhsprogram.com/pubs/pdf/FR308/FR308.pdf. Accessed May 2016.

[21] Development Partners for Health in Kenya. Kenya health facilities. 2016. http://dphk.or.ke/. Accessed June 2016.

[22] Government of Kenya, Ministry of Health (GoK, MoH). DHIS2: Kenya Health Information System. https://hiskenya.org/. Accessed June 2016.

[23] Google Earth: https://www.google.com/earth/. Accessed Apr 2016.

[24] OpenStreetMap: http://www.openstreetmap.org/. Accessed Apr 2016.

[25] Ministry of Health-Government of Kenya. Kenya Health Policy 2014–2030. 2014. http://www.cickenya.org/index.php/policies-regulations/item/373-kenya-health-policy-2012-2030#.WCP7ncnLyPU. Accessed May 2016.

[26] Jun C, Ban Y, Li S (2014). China: open access to Earth land-cover map. Nature.

[27] USGS Earth Explorer: https://earthexplorer.usgs.gov/. Accessed May 2016.

[28] Ministry of Transport-Government of Kenya. Kenya National Highways Authority. Kenya Roads Map. 2014.

[29] Worldpop: http://www.worldpop.org.uk/data/. Accessed May 2016.

[30] Blanford JI, Kumar S, Luo W, MacEachren AM (2012). It’s a long, long walk: accessibility to hospitals, maternity and integrated health centers in Niger. Int J Health Geogr.

[31] Demidenko E (2013). Mixed models: theory and applications with R.

[32] Bates DM, Maechler M, Bolker B, Walker S (2015). Fitting linear mixed-effects models using lme4. J Stat Softw.

[33] Oliver MA, Webster R (2014). A tutorial guide to geostatistics: computing and modelling variograms and kriging. Catena.

[34] Hanley JA, McNeil BJ (1982). The meaning and use of the area under a receiver operating (ROC) curvel characteristic. Radiology.

[35] Mousavi SZ, Kavian A, Soleimani K, Mousavi SR, Shirzadi A (2011). GIS-based spatial prediction of landslide susceptibility using logistic regression model. Geomat Nat Hazards Risk.

[36] Florkowski CM (2008). Sensitivity, specificity, receiver-operating characteristic (ROC) curves and likelihood ratios: communicating the performance of diagnostic tests. Clin Biochem Rev.

[37] Ministry of Health-Government of Kenya. Kenya Household Health Expenditure and Utilisation Survey 2013. http://www.healthpolicyproject.com/pubs/745_KHHUESReportJanuary.pdf. Accessed Sept 2016.

