# Supplementary material for: Univariate and multivariate spatial models of health facility utilisation for childhood fevers in an area on the coast of Kenya
Source: Int J Health Geogr. 2017 Sep 18;16:34. doi: 10.1186/s12942-017-0107-7 (PMC5604359; doi:10.1186/s12942-017-0107-7)
Supplement: Supplementary file 1 — Additional file 1. Additional file on the determinats of health facility utilisation and detailed kriging methods used for interpolation of covariates. [file 12942_2017_107_MOESM1_ESM.docx]

**Additional file 1**

**Univariate and multivariate spatial models of health facility utilisation for childhood fevers in an area on the coast of Kenya**

**Paul O Ouma^1,2*^, Nathan O Agutu^1^, Robert W Snow^2,3^ and Abdisalan M Noor^2,3^**

1. Jomo Kenyatta University of Agriculture and Technology, Department of Geomatic Engineering and Geospatial Information Systems, Nairobi, Kenya
2. Kenya Medical Research Institute/Wellcome Trust Research Programme, Nairobi, Kenya
3. Centre for Tropical Medicine and Global Health, Nuffield Department of Clinical Medicine, University of Oxford, UK.

**Table of Contents**

[1 Determinants of treatment seeking for fever 2](#_Toc483317892)

[2 Spatial prediction of variables 3](#_Toc483317893)

[3 Comparing both utilisation models using the percent correct prediction test 7](#_Toc483317894)

# Determinants of treatment seeking for fever

In a study to identify treatment seeking patterns for childhood illnesses [1], belonging to a higher household income level or **wealth** quintile increased the chances of seeking treatment at a health facility. Similar results are observed elsewhere [2–6], where children from wealthier households were likely to seek recommended treatment for illnesses. This is because in the poorer households, there is increasing burden experienced when paying for healthcare or using transport services when seeking healthcare [7–11]. A study that looked at the broader perspective of treatment seeking patterns among different socio-economic groups found that there were greater differences in treatment seeking patters among different SES groups in urban areas. The urban poor were experiencing greater challenges compared to the wealthier urban groups [11].

The opposite relationship has also been observed, where coming from a wealthier household reduced the chances of seeking treatment at recommended service providers [12,13]. One of the explanations for this is that in many cases, the clinical symptoms used to assess relationships are more prevalent among the poorer than in the richer households.

**Maternal education** is also commonly mentioned as a factor affecting health facility utilization. All studies reviewed indicated that increasing education of the family decision makers is associated with increased probability of seeking treatment [1,14–17]. This is due to the increased knowledge of healthcare needs, sanitation and healthy habits among the more educated population sub groups. The presence of health issues in standard educational curricular is pointed out as one the reason for increased appreciation of the need to access and use health facilities for illnesses. **Age** is also a significant factor associated with treatment seeking as defined in [18,19].

A much more qualitative factor affecting health facility utilization is **severity of the disease** [3,6,7,10,15,18,20,21]**.** In all the studies reviewed, utilization of health facilities is higher in groups where severity is perceived to be higher. The explanation for this is that in general, people use what is available to them, but willingness to make a greater effort to seek formal healthcare increases with increasing severity of the disease. Probably the challenge with recording severity of illness is that perception varies. This challenge is much more elevated if the respondents are guardians of children, with studies showing that caregivers are much more likely to differentiate mild from severe illnesses [19].

**Accessibility** The relationship of access to health facilities with utilization for fever/malaria is aptly captured in many studies [1,9,14,19,22–31]. As a major component of accessibility, physical access is normally used with a near universal consensus that increasing travel time to health facilities reduces the probability of seeking formal health services (“*Decay effect*”). This is more pronounced in rural areas where the physical separation between populations and service providers are much more enhanced [32].

Other demographic characteristics within the households can also affect the decision whether to attend or not attend a health facility. These include ethnicity, the family decision maker, competing household priorities, household sizes [25] and the occupation of the bread winner. Health facility characteristics such as the level of care have also been identified as possible supply side factors [33]. Other studies have also demonstrated the influence of perceived service quality [9,34], with services thought to be having poor quality such as lower level facilities being less used [27]. Finally, inequity in access has also been observed in different communities with similar characteristics [35] and also in different residences such as rural and urban [41]. These are important demand side barriers towards treatment seeking for common illnesses. Provider characteristics such as effectiveness of service delivery and different levels of care are also emerging as important, when analysing access in general [36].

Determinants of health facility utilisation were either individual, household or cluster characteristics. At individual level, we considered use of insecticide treated net (ITN) the previous night, and age group of the children in terms of years. At house-hold level, those considered were mother’s education, number of children in a household, wealth index and access to improved sanitation. At cluster level, urbanisation and travel time to health facilities was used. Selection of variables was restricted to those that we thought could be spatially modelled.

# Spatial prediction of variables

We used Kriging to spatially interpolate wealth and household number of children. The kriging equation is given by;

Where u refers to a location, $Z^{*}(\mathrm{u})$ is an estimate at location u, with n data values. $\lambda_{i}$ Refer to the kriging weights. Matheron in 1962, introduced the concept of semivariances which are used to estimate kriging weights [37];

$$\gamma(\mathrm{h})=\frac{1}{2}E[(\mathrm{z}(\mathrm{s}_{i})-\mathrm{z}(\mathrm{s}_{i}+\mathrm{h}))^{2}]$$

Where $\mathrm{z}(\mathrm{s}_{i})$is the target value at a sampled location and $\mathrm{z}(\mathrm{s}_{i}+\mathrm{h})$is the value at a neighboring location with a distance $\mathrm{s}_{i}+\mathrm{h}$from the target point. In this case, we have n sampled locations, this yields $\mathrm{n}\cdot(\mathrm{n}-1)/2$ pairs which will be used for estimating the semivariance.

The semivariances can be plotted against their corresponding distances (*lag*) to produce a variogram cloud. If a spatial structure exists for the sampled data points then we expect to see smaller semivariances for smaller distances, with variances increasing with increasing distance (lag). The interpretation is that observed values at shorter distances are much more similar, with this ‘*similarity*’ reducing as distance increases, up to a certain distance (*sill*) beyond which differences between any two pair of points tends to be equal to the global variance [38]. Figure 3.11 semivariogram models for the variables used.


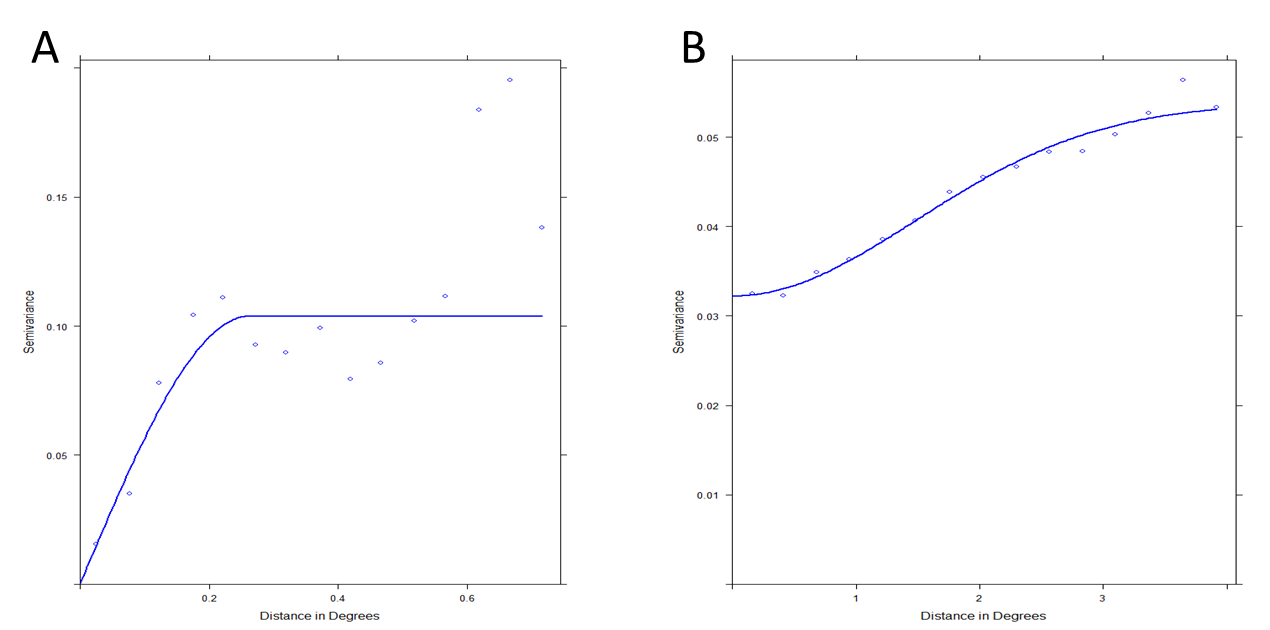


Figure 1 Modelled semivariograms for the different variables of interest. The x axes show the distance in degrees while the y axes show the semivariances for each of the variables. A) Is for Poverty and shows less variation past 0.23 degrees (26 km. The model B) shows significant variation of households with <=1 child up until about 3 degrees (340 km).

**Ordinary kriging formulation**

The equations and formulations for ordinary kriging are given below;

Let $z(\mathrm{s}_{i}),i=1,2,.....,N$be the observed values of a variable s at points **s_1_,s_2_,…..,s_N_,** which are defined in a two dimension space $S_{i}\equiv\{\mathrm{s}_{i1},\mathrm{s}_{i2}\}^{T}$. For any new point, **x_0_**we wish to predict Z as;

$$\hat{Z}(\mathbf{s}_{\mathbf{0}})=\sum_{i=1}^{N} \lambda_{i}Z(\mathbf{s}_{\mathbf{i}})$$

$\lambda_{i},i=1,2,....,N$are the weights which are chosen in order to ensure minimal prediction error variance by solving equation;

$\sum_{i=1}^{N} \lambda_{i}\gamma(\mathbf{s}_{\mathbf{i}}\mathbf{-s}_{\mathbf{j}})+\psi(\mathbf{s}_{\mathbf{0}})=\gamma(\mathbf{s}_{\mathbf{j}}\mathbf{-s}_{\mathbf{0}})$for all *j*

We also know that $\sum_{i=1}^{N} \lambda_{i}=1$

Here, $\gamma(\mathrm{s}_{i}-s_{j})$ is the semivariance between data points *i* and *j,* $\gamma(s_{j}-s_{0})$ is the semivariance between data point *j* and the target point $s_{0}$, $\psi(\mathrm{s}_{0})$term is the Lagrange multiplier introduced to minimize the error variance.

In matrix notation, this is given as; $\mathbf{A\lambda=b}$. Calculating the inverse of $\mathbf{A}$and multiplying the result by $\mathbf{b}$gives the weights $\lambda\equiv\{\lambda_{1},\lambda_{2}.......,\lambda_{N}\}$ which if inserted into equation (7) we get the predictions.

The error variance of the prediction is given by;

$$\sigma^{2}(\mathbf{s}_{\mathbf{0}})=\mathbf{b}^{\mathbf{T}}\mathbf{\lambda}$$

Predictions for any points of the area are still the weighted sum of the data:

$\hat{Z}(\mathrm{B})=\sum_{i=1}^{N} \lambda_{i}Z(\mathbf{s}_{\mathbf{i}})$with the kriging system solved as;

$$\sum_{i=1}^{N} \lambda_{i}\gamma(\mathbf{s}_{\mathbf{i}}\mathbf{-s}_{\mathbf{j}})+\psi(\mathrm{B})=\bar{\gamma}(\mathbf{s}_{\mathbf{j}},\mathrm{B})$$

Where $\bar{\gamma}(\mathbf{s}_{\mathbf{j}},\mathrm{B})$is the average semivariance between the data and the target block and **b** now is the right-hand sides of Equation.

The geoR package in R v3.0 statistical software was used to perform the prediction [39]. All the predictions were carried out at 300m spatial resolution at country level and the study area extracted from the predictions. This resolution was used due to the time required in modelling but the predictions were resampled to 100m for the subsequent analysis.

**Predicted outcomes**


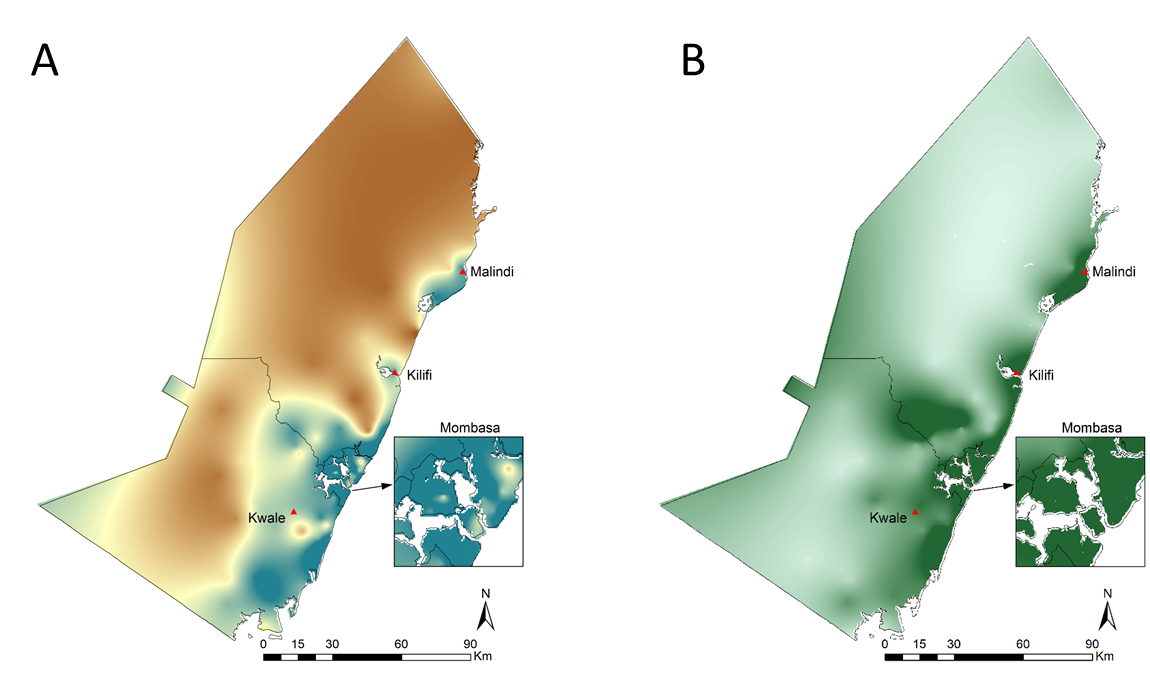


Figure 2 the proportion of population living in poverty was lowest in urban areas around Mombasa, Kilifi and Malindi. Probability of finding households with more than 1 child under 5 was highest around Mombasa and areas around Kwale and Malindi towns.

# Comparing both utilisation models using the percent correct prediction test

The sensitivity analysis of this accuracy assessment using different cut off values are shown in Table 2.

Table 2 result of sensitivity analysis

| **Cut off value** | **Model 1 (Time)** | **Model 2 (covariates)** |
| --- | --- | --- |
| 0.50 | 53% | 61% |
| 0.55 | 45% | 61% |
| 0.60 | 45% | 61% |
| 0.45 | 53% | 53% |
| 0.40 | 53% | 53% |

**References**

1. Kanté AM, Gutierrez HR, Larsen AM, Jackson EF, Helleringer S, Exavery A, et al. Childhood Illness Prevalence and Health Seeking Behavior Patterns in Rural Tanzania. BMC Public Health; 2015;15:951.

2. Diaz T, George AS, Rao SR, Bangura PS, Baimba JB, McMahon S a, et al. Healthcare seeking for diarrhoea, malaria and pneumonia among children in four poor rural districts in Sierra Leone in the context of free health care: results of a cross-sectional survey. BMC Public Health; 2013;13:157.

3. Burton DC, Flannery B, Onyango B, Larson C, Alaii J, Zhang X, et al. Healthcare-seeking behaviour for common infectious disease-related illnesses in rural Kenya: A community-based house-to-house survey. J Heal Popul Nutr. 2011;29:61–70.

4. Ellis AA, Traore S, Doumbia S, Dalglish SL, Winch PJ. Treatment actions and treatment failure: case studies in the response to severe childhood febrile illness in Mali. BMC Public Health; 2012;12:946.

5. Rutebemberwa E, Kallander K, Tomson G, Peterson S, Pariyo G. Determinants of delay in care-seeking for febrile children in eastern Uganda. Trop Med Int Heal. 2009;14:472–9.

6. Taffa N, Chepngeno G. Determinants of health care seeking for childhood illnesses in Nairobi slums. Trop Med Int Heal. Heal. 2005;10:240–5.

7. Nyamongo IK. Health care switching behaviour of malaria patients in a Kenyan rural community. Soc Sci Med. 2002;54:377–86.

8. Mwenesi H, Harpham T, Snow RW. Child malaria treatment practices among mothers in Kenya. Soc Sci Med. 1995;40:1271–7.

9. Mbonye AK. Prevalence of childhood illnesses and care-seeking practices in rural Uganda. ScientificWorldJournal. 2003;3:721–30.

10. Chuma J, Abuya T, Memusi D, Juma E, Akhwale W, Ntwiga J, et al. Reviewing the literature on access to prompt and effective malaria treatment in Kenya: implications for meeting the Abuja targets. Malar J. 2009;8:243.

11. Chuma J, Gilson L, Molyneux C. Treatment-seeking behaviour , cost burdens and coping strategies among rural and urban households in Coastal Kenya : an equity analysis. Trop Med Int Heal. 2007;12:673–86.

12. Ibe OP, Mangham-Jefferies L, Cundill B, Wiseman V, Uzochukwu BS, Onwujekwe OE. Quality of care for the treatment for uncomplicated malaria in South-East Nigeria: how important is socioeconomic status? Int J Equity Health. 2015;14:1–9.

13. Novignon J, Nonvignon J. Socioeconomic status and the prevalence of fever in children under age five: evidence from four sub-Saharan African countries. BMC Res Notes. BMC Research Notes; 2012;5:380.

14. Das A, Ravindran TS. Factors affecting treatment-seeking for febrile illness in a malaria endemic block in Boudh district, Orissa, India: policy implications for malaria control. Malar J. 2010;9:377.

15. Webair HH, Bin-Gouth AS. Factors affecting health seeking behavior for common childhood illnesses in Yemen. Patient Prefer. Adherence. 2013;7:1129–38.

16. Kazembe LN, Appleton CC, Kleinschmidt I. Choice of treatment for fever at household level in Malawi: examining spatial patterns. Malar. J. 2007;6:40.

17. Hwang J, Graves PM, Jima D, Reithinger R, Patrick Kachur S. Knowledge of malaria and its association with malaria-related behaviors - Results from the Malaria Indicator Survey, Ethiopia, 2007. PLoS One. 2010;5.

18. Ustrup M, Ngwira B, Stockman LJ, Deming M, Nyasulu P, Bowie C, et al. Potential barriers to healthcare in malawi for under-five children with cough and fever: A national household survey. J Heal Popul Nutr. 2014;32:68–78.

19. Ewing VL, Lalloo DG, Phiri KS, Roca-Feltrer A, Mangham LJ, SanJoaquin M a. Seasonal and geographic differences in treatment-seeking and household cost of febrile illness among children in Malawi. Malar J. BioMed Central Ltd; 2011;10:32.

20. Chibwana AI, Mathanga DP, Chinkhumba J, Campbell CH. Socio-cultural predictors of health-seeking behaviour for febrile under-five children in Mwanza-Neno district, Malawi. Malar J. 2009;8:219.

21. Salah MT, Adam I, Malik EM. Care-seeking behavior for Fever in children under five years in an urban area in eastern Sudan. J Fam Community Med. 2007;14:25–8.

22. Okeke TA, Okeibunor JC. Rural-urban differences in health-seeking for the treatment of childhood malaria in south-east Nigeria. Health Policy. 2010;95:62–8.

23. Kassile T, Lokina R, Mujinja P, Mmbando BP. Determinants of delay in care seeking among children under five with fever in Dodoma region, central Tanzania: a cross-sectional study. Malar J. 2014;13:348.

24. Das A, Das Gupta RK, Friedman J, Pradhan MM, Mohapatra CC, Sandhibigraha D. Community perceptions on malaria and care-seeking practices in endemic Indian settings: policy implications for the malaria control programme. Malar J. 2013;12:39.

25. Bigogo G, Audi A, Aura B, Aol G, Breiman RF, Feikin DR. Health-seeking patterns among participants of population-based morbidity surveillance in rural western Kenya: Implications for calculating disease rates. Int J Infect Dis. 2010;14:e967–73.

26. Nonvignon J, Aikins MKS, Chinbuah MA, Abbey M, Gyapong M, Garshong BNA, et al. Treatment choices for fevers in children under-five years in a rural Ghanaian district. Malar J. 2010;9:188.

27. Mbagaya GM, Odhiambo MO, Oniang’o RK. Mother’s health seeking behaviour during child illness in a rural Western Kenya community. Afr Health Sci. 2005;5:322–7.

28. Krumkamp R, Sarpong N, Kreuels B, Ehlkes L, Loag W, Schwarz NG, et al. Health care utilization and symptom severity in Ghanaian children - A cross-sectional study. PLoS One. 2013;8:1–7.

29. Meara WPO, Noor A, Gatakaa H, Tsofa B, Mckenzie FE, Marsh K. The impact of primary health care on malaria morbidity – defining access by disease burden. 2009;14:29–35.

30. Feikin DR, Nguyen LM, Adazu K, Ombok M, Audi A, Slutsker L, et al. The impact of distance of residence from a peripheral health facility on pediatric health utilisation in rural western Kenya. Trop. Med. Int. Heal. 2009;14:54–61.

31. Noor AM, Amin A a., Gething PW, Atkinson PM, Hay SI, Snow RW. Modelling distances travelled to government health services in Kenya. Trop Med Int Heal. 2006;11:188–96.

32. Noor AM, Zurovac D, Hay SI, Ochola SA, Snow RW. Defining equity in physical access to clinical services using geographical information systems as part of malaria planning and monitoring in Kenya. Trop Med Int Heal. 2003;8:917–26.

33. Kahabuka C, Kvåle G, Hinderaker SG. Care-Seeking and Management of Common Childhood Illnesses in Tanzania - Results from the 2010 Demographic and Health Survey. PLoS One. 2013;8.

34. Rutebemberwa E, Pariyo G, Peterson S, Tomson G, Kallander K. Utilization of public or private health care providers by febrile children after user fee removal in Uganda. Malar. J. [Internet]. 2009;8:45.

35. Odu BP, Mitchell S, Isa H, Ugot I, Yusuf R, Cockcroft A, et al. Equity and seeking treatment for young children with fever in Nigeria: a cross-sectional study in Cross River and Bauchi States. Infect Dis Poverty. 2015;4:1.

36. Kizito J, Kayendeke M, Nabirye C, Staedke SG, Chandler CIR. Improving access to health care for malaria in Africa: a review of literature on what attracts patients. Malar J. 2012;11:55.

37. Hengl T. A Practical guide to Geostatistical Mapping. First Edit. Sci. Tech. Res. Ser. 2009.

38. Cressie NAC. Statistics for Spatial Data. Comput. Stat. Data Anal. 1993;14:547.

39. Ribeiro PJ, Diggle PJ. Analysis of geostatistical data. 2009;1–83.
